# Cyb5r3 activation rescues secondary failure to sulfonylurea but not β-cell dedifferentiation

**DOI:** 10.1371/journal.pone.0297555

**Published:** 2024-02-09

**Authors:** Hitoshi Watanabe, Shun-ichiro Asahara, Jinsook Son, Wendy M. McKimpson, Rafael de Cabo, Domenico Accili

**Affiliations:** 1 Department of Medicine, Vagelos College of Physicians and Surgeons, Columbia University, New York, New York, United States of America; 2 Naomi Berrie Diabetes Center, Vagelos College of Physicians and Surgeons, Columbia University, New York, New York, United States of America; 3 Division of Diabetes and Endocrinology, Department of Internal Medicine, Kobe University Graduate School of Medicine, Kobe, Japan; 4 Translational Gerontology Branch, National Institute on Aging, National Institutes of Health, Baltimore, Maryland, United States of America; International University of Health and Welfare School of Medicine, JAPAN

## Abstract

Diabetes mellitus is characterized by insulin resistance and β-cell failure. The latter involves impaired insulin secretion and β-cell dedifferentiation. Sulfonylurea (SU) is used to improve insulin secretion in diabetes, but it suffers from secondary failure. The relationship between SU secondary failure and β-cell dedifferentiation has not been examined. Using a model of SU secondary failure, we have previously shown that functional loss of oxidoreductase Cyb5r3 mediates effects of SU failure through interactions with glucokinase. Here we demonstrate that SU failure is associated with partial β-cell dedifferentiation. Cyb5r3 knockout mice show more pronounced β-cell dedifferentiation and glucose intolerance after chronic SU administration, high-fat diet feeding, and during aging. A Cyb5r3 activator improves impaired insulin secretion caused by chronic SU treatment, but not β-cell dedifferentiation. We conclude that chronic SU administration affects progression of β-cell dedifferentiation and that Cyb5r3 activation reverses secondary failure to SU without restoring β-cell dedifferentiation.

## Introduction

Type 2 diabetes is caused by β-cell dysfunction and insulin resistance. Sulfonylurea (SU) potently induce insulin secretion and are prescribed widely for type 2 diabetes treatment [1]. SU have been used for the management of type 2 diabetes since the 1950s and are still widely used not only in low- and middle-income countries, but also in developed countries because of their efficacy and low cost [2, 3]. On the other hand, their use is curtailed by secondary failure, risk of hypoglycemia, weight gain, and concerns about cardiovascular safety [3]. Chronic treatment with SU is associated with a secondary failure rate of 5–10% per year [4–6]. In the UKPD study, after 6 years of treatment with sulfonylurea, 30% of patients required combination therapy (20% with metformin and 10% with insulin) [7], as did 34% of patients in the ADOPT study [8]. The mechanisms of secondary failure in pancreatic β-cells are not fully understood.

We have recently described a mechanism of SU failure that links the cell homeostatic function of FoxO1 and its target gene, oxidoreductase cytochrome b5 reductase 3 (Cyb5r3) [9]. Cyb5r3 has pleiotropic cellular functions, including as a component of the mitochondrial electron transfer system [10, 11]. We discovered that Cyb5r3 regulates glucose utilization in β-cells by increasing the stability of glucokinase, the rate-limiting enzyme for glycolysis. Chronic treatment with glibenclamide (GLB), a representative second generation long-acting sulfonylurea, decreases FoxO1 and Cyb5r3 levels in murine as well as human islets. As a consequence, glycolysis is impaired and insulin secretion decreased. Cyb5r3 activation can rescue SU secondary failure following chronic GLB treatment in mouse and human islets, making Cyb5r3 a potential therapeutic target to extend SU utilization and restore insulin secretion [9].

FoxO1 orchestrates complex β-cell homeostatic functions [12]. Quiescent in resting β-cells, it becomes activated through nuclear translocation in response to hyperglycemia [13], elevations of fatty acids [14], and cytokines [15]. Activation leads to increased turnover and reduced levels [13, 16]. This process is associated with defective insulin secretion [17], altered mitochondrial substrate preferences [10], and β-cell dedifferentiation [16]. Experimental animal studies and ex vivo studies in human diabetic islets have shown that dedifferentiated β-cells can be restored to a normal function by nutritional and pharmacologic methods, indicating that β-cell dedifferentiation is reversible [18, 19]. In contrast, treatment with insulin secretagogues such as SU exacerbates β-cell failure.

Given that chronic SU treatment decreases FoxO1 levels, a process that leads to β-cell dedifferentiation, we asked whether improvement of SU secondary failure by activation of Cyb5r3 also improves β-cell dedifferentiation. To answer this question, we assessed (i) the effect of chronic GLB treatment on β-cell dedifferentiation, (ii) the progression of β-cell dedifferentiation in Cyb5r3 β-cell-specific knockout mice (Cyb5r3 βKO), and (iii) the effect of tetrahydroindenoindole (THII), a potential activator of Cyb5r3, on β-cell function and dedifferentiation.

The main new finding of our study is the dissociation of β-cell dedifferentiation–as measured by activation of Aldehyde dehydrogenase 1 isoform A3 (Aldh1a3)–from SU-induced insulin secretory failure. As we have shown in prior work, β-cell dedifferentiation is preceded by impaired insulin secretion, consistent with the evolving pathophysiology of diabetes, characterized by an early and reversible impairment of insulin secretion. Interestingly, this process is exacerbated by SU treatment. Thus, the question addressed in this paper is whether secondary failure to SU is already a sign of dedifferentiation or not. The data show that it is not, and are consistent with our recent demonstration that beta cell dedifferentiation can be separated into distinct sub-phenotypes, each one of which has its own mediators.

## Materials and methods

### Animals

Mice were fed standard chow (PicoLab rodent diet 20, 5053; Purina Mills) and maintained in a climate-controlled room on a 12-h light/dark cycle with lights on at 7:00 AM and off at 7:00 PM. C57BL/6 mice were from The Jackson Laboratories (Bar Harbor, ME). Cyb5r3 βKO have been described [11]. Eight- to 12-week-old mice were subjected to drug treatment with a GLB-filled (20 mg/kg/day) (Sigma, St. Louis, MO) osmotic pump (ALZET, Cupertino, CA) intraperitoneally for 6 weeks. THII (100 μM) was administered in drinking water ad libitum for 6 weeks at the same time as GLB treatment. As a model of obesity, mice were fed a high-fat diet (HFD) (D12492; Research Diet Inc., New Brunswick, NJ) for 20 weeks. For aging studies, we used standard chow-fed 90- to 110-week-old mice. CO2 euthanasia was performed for sample collection in accordance with the AVMA Guidelines for Animal Euthanasia (2020 Edition). Columbia University Institutional Animal Care and Use Committee approved all experiments according to NIH guidelines and all experiments were performed following relevant guidelines and regulations and in compliance with the ARRIVE guidelines (protocol number AAAS9451).

### Immunohistochemistry

Tissues were fixed with 4% paraformaldehyde/PBS for 16 h at 4°C, replaced with 30% sucrose for 24 h, embedded in OCT compound (Sakura Finetek Inc, Tokyo, Japan), and cut into 7 μm-thick frozen sections. We used anti-Aldh1a3 (NBP2-15339; Novus Biologicals, Littleton, CO), anti-insulin (IR00261-2; Dako, Carpinteria, CA), and anti-Pdx1 (5679S; Cell Signaling Technology, Danvers, MA) antibodies as primary, and Alexa Fluor–conjugated as secondary antibodies (ThermoFisher, Waltham, MA). We used confocal microscopy and Laser Scanning Microscope Software (Zeiss LSM 510 and 710) to capture images [20].

### RNA analysis

We used RNeasy Mini Kit (Qiagen, Venlo, Netherlands) to extract total RNA from isolated islets and qScript cDNA Synthesis Kit (QuantaBio, Beverly, MA) to synthesize cDNA. Quantitative real-time PCR was performed with GoTaq qPCR Master Mix (Promega) on a Bio-Rad CFX96 real-time PCR system. Gene expression levels were normalized by HPRT expression as internal control. The primer sequences are as follows: Hprt, forward 5’- TGA TCA GTC AAC GGG GGA CA-3’ and reverse 5’- TTC GAG AGG TCC TTT TCA CCA-3’; FoxO1, forward 5’- TCC AGT TCC TTC ATT CTG CAC T-3’ and reverse 5’- GCG TGC CCT ACT TCA AGG ATA A-3’; Cyb5r3, forward 5’- ACA TCC TGG GCC TTC CTA TT-3’ and reverse 5’-TTG ACC ACC AAG TCC ACA AA-3’; MafA, forward 5’-GTC ATC CGA CTG AAA CAG AA-3’ and reverse 5’-GCC AAC TTC TCG TAT TTC TC-3’; Pdx1, forward 5’-CCC CAG TTT ACA AGC TCG CT-3’ and reverse 5’-CTC GGT TCC ATT CGG GAA AGG-3’; Neurogenin 3, forward 5’-TGG CCC ATA GAT GAT GTT CG-3’ and reverse 5’-AGA AGG CAG ATC ACC TTC GTG-3’; Ins1, forward 5’- CCC CAC CTG GAG ACC TTA AT-3’ and reverse 5’- TGA AAC AAT GAC CTG CTT GC-3’; Ins2, forward 5’- GGA GCA GGT GAC CTT CAG AC-3’ and reverse 5’- AGA GGG GTA GGC TGG GTA GT-3’; Kir6.2, forward 5’- AGA ATA TCG TCG GGC TGA TG-3’ and reverse 5’- CTC TTT CGG AGG TCC CCT AC-3’; Sur1, forward 5’- TGA AGC AGA CC AAC GAG ATG-3’ and reverse 5’- GCA ATG GGG ATA GCT GTG TT-3’.

### Protein analysis

Islets were homogenized in ice cold CelLytic MT Cell Lysis Reagent (Sigma, St. Louis, MO) with protease and phosphatase inhibitors (Cell Signaling Technology, Danvers, MA). We used anti-Aldh1a3 (NBP2-15339; Novus Biologicals) and anti-β-actin antibodies (#3770, Cell Signaling Technology) for immunoblotting, and Odyssey imaging system (LI-COR, Lincoln, NE) to obtain images.

### Metabolic analysis

We performed glucose tolerance test (2 g/kg) after a 16-h fast. Blood samples were obtained from the tail vein. Blood glucose level was measured using a CONTOUR NEXT ONE (Ascensia Diabetes Care, Valhalla, NY). Plasma insulin was measured by ELISA (Mercodia, Winston Salem, NC).

### Statistical analyses

Statistical analyses were performed using Prism software (Graph Pad). Data are presented as mean ± SEM. Statistical analysis was performed by Student’s t-test for comparison between two groups and two-way ANOVA followed by Tukey’s test for effects of two variables. Differences were considered significant at *P*< 0.05.

## Results

### Chronic GLB causes partial dedifferentiation and impairs insulin secretion

**Fig 1:**
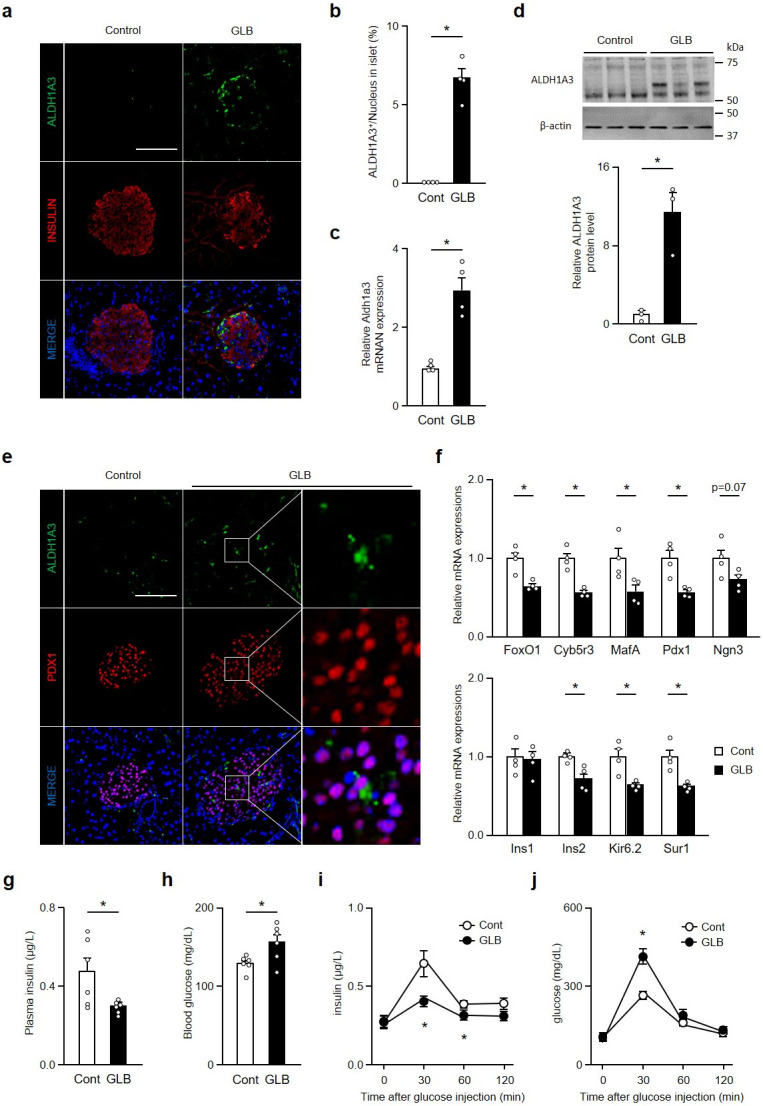
Chronic GLB treatment increases marker of β-cell dedifferentiation. a: Representative Aldh1a3 staining (green) in islets from pancreatic sections of mice treated with GLB for 6 weeks (n = 4). Scale bars: 50 μm. b-d: Quantification of Aldh1a3 positive cells (B), Aldh1a3 mRNA expression (c) and Aldh1a3 protein levels (d) in islets from treated with GLB (n = 4). e: Representative images from pancreatic sections of mice treated with GLB stained with Aldh1a3 (green), Pdx1 (red), and DAPI (blue). Scale bars: 50 μm. f: Relative mRNA expressions of FoxO1, Cyb5r3, MafA, Pdx1, Ngn3, Ins1, Ins2, Kir6.2 and Sur1 in islets from mice treated with GLB (n = 4). g and h: Plasma insulin (g) and blood glucose (h) levels in mice treated with GLB under free feeding condition (n = 4–6). i and j: Plasma insulin (i) and blood glucose (j) levels in mice treated with GLB during glucose tolerance test (n = 4). *P < 0.05 by unpaired Student’s t-test. Data represent means ± SEM.

We have shown that GLB treatment for 3–4 weeks causes SU secondary ineffectiveness and decreases SU-induced insulin secretion [9]. To determine whether chronic GLB administration induces β-cell dedifferentiation, we treated mice with GLB for 6 weeks and assessed Aldh1a3-positive cells, a marker of β-cell dedifferentiation (Fig 1a) [21, 22]. Aldh1a3-positive cells were present in islets from mice treated with GLB at a rate of ~ 5% (Fig 1b). Aldh1a3 mRNA and protein levels in isolated islets from mice treated with GLB also increased (Fig 1c and 1d). We performed Aldh1a3 and Pdx1 immunohistochemistry to determine if Aldh1a3-positive cells were β-cells. All Aldh1a3-positive cells were Pdx1-positive (Fig 1e). As FoxO1 is involved in β-cell dedifferentiation, we measured expression of FoxO1 and its target gene Cyb5r3, which regulates insulin secretion. GLB treatment decreased FoxO1 and Cyb5r3 mRNA (Fig 1f). In addition, expression of β-cell markers MafA, Pdx1, Ins2, Kir6.2 and Sur1 were decreased in the GLB-treated group. To investigate glucose-induced insulin secretion in GLB-treated mice, we measured plasma insulin and blood glucose levels under free-feeding conditions and during glucose tolerance tests. Under free-feeding condition, plasma insulin levels were lower and glucose levels higher in GLB-treated mice compared with controls (Fig 1g and 1h). GLB-treated mice showed decreased glucose-induced insulin secretion and glucose intolerance (Fig 1i and 1j). These data suggest that chronic GLB treatment induces β-cell dedifferentiation as well as decreased β-cell function.

### β-cell Cyb5r3 knockout impairs insulin secretion and predisposes to β-cell dedifferentiation

**Fig 2:**
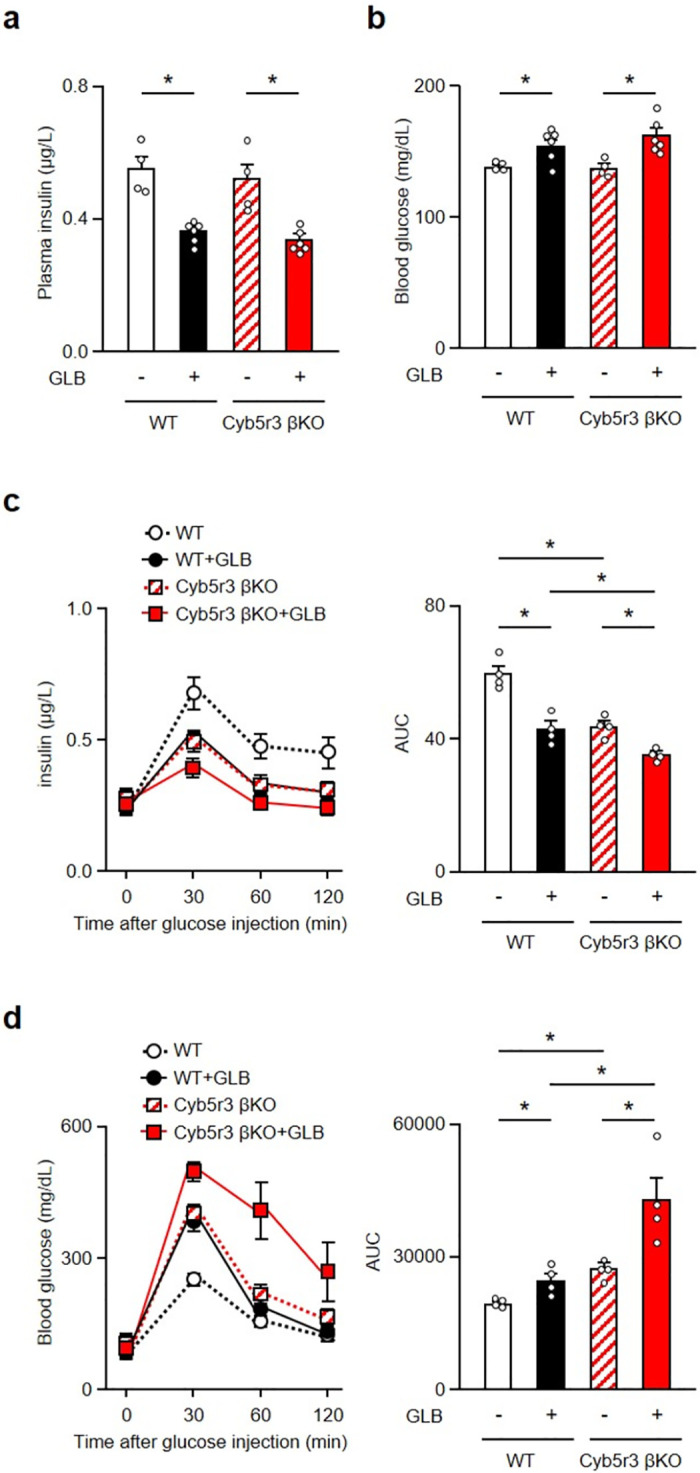
Chronic GLB treatment further impairs glucose-induced insulin secretion in Cyb5r3 βKO. a and b: Plasma insulin (a) blood glucose (b) levels in WT and Cyb5r3 βKO treated with GLB under free feeding condition (n = 4). c and d: Plasma insulin level and its area under the curve (c) and blood glucose levels and its area under the curve (d) in WT and Cyb5r3 βKO treated with GLB during glucose tolerance test (n = 4). *P < 0.05 by two-way ANOVA followed by Tukey’s test. Data represent means ± SEM.

We have shown that chronic GLB administration decreased Foxo1 and its target gene Cyb5r3 expression in pancreatic islets [9]. Cyb5r3 plays a role in the mechanism of SU secondary failure. To determine whether Cyb5r3 is involved in β-cell dedifferentiation due to loss of FoxO1, we measured glucose-induced insulin secretion and β-cell dedifferentiation in Cyb5r3 βKO treated with GLB for six weeks. Plasma insulin levels were similarly decreased, and glucose levels increased by GLB treatment under free feeding conditions in both wild type (WT) mice and Cyb5r3 βKO (Fig 2a and 2b). During glucose tolerance tests, insulin levels were reduced by GLB administration, more so in Cyb5r3 βKO than WT mice (Fig 2c). GLB administration to Cyb5r3 βKO had additive effects to decrease insulin levels. GLB-treated and Cyb5r3 βKO impaired glucose tolerance compared to WT mice. GLB administration to Cyb5r3 βKO further deteriorated glucose tolerance (Fig 2d).

**Fig 3:**
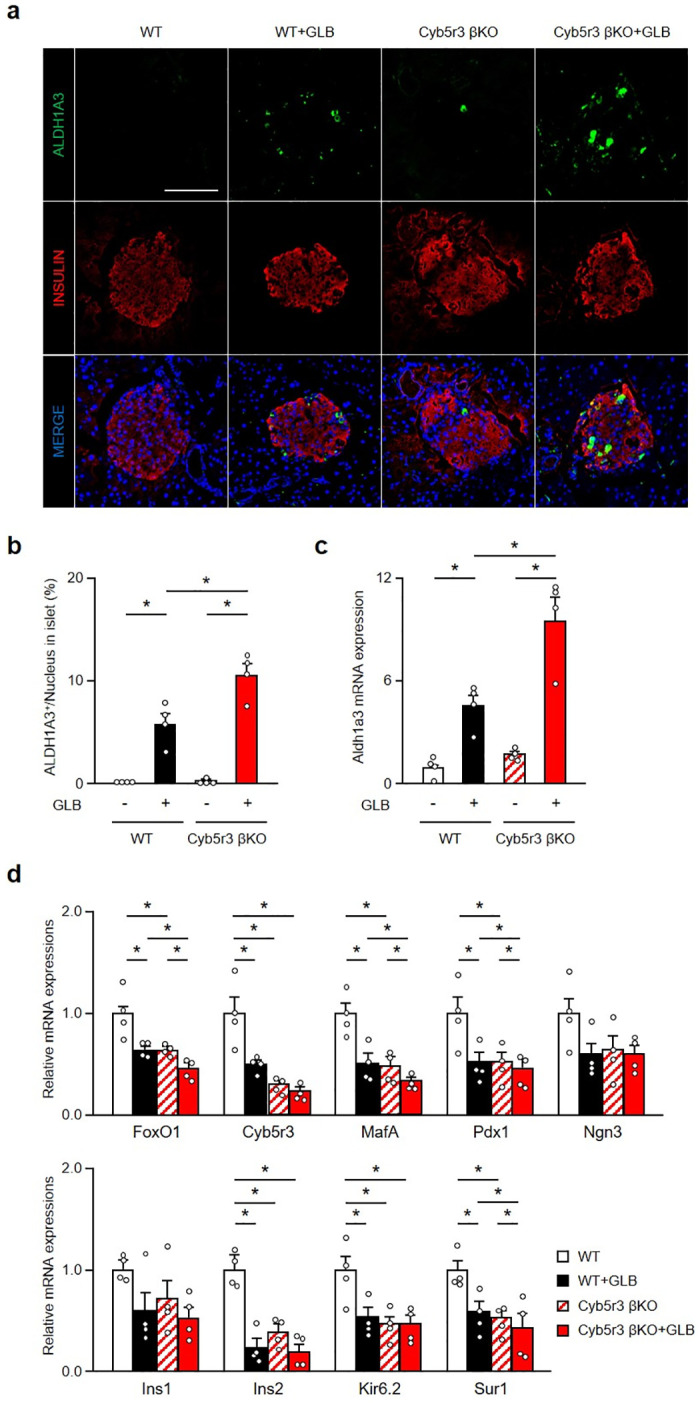
Cyb5r3 deletion increases β-cell dedifferentiation by chronic GLB treatment. a: Representative Aldh1a3 staining (green) in islets from pancreatic sections of WT and Cyb5r3 βKO treated with GLB for 6 weeks (n = 4). Scale bars: 50 μm. b and c: Quantification of Aldh1a3 positive cells (b) and Aldh1a3 mRNA expression (c) in islets from WT and Cyb5r3 βKO treated with GLB (n = 4). d: Relative mRNA expressions of FoxO1, Cyb5r3, MafA, Pdx1, Ngn3, Ins1, Ins2, Kir6.2 and Sur1 in islets from WT and Cyb5r3 βKO treated with GLB (n = 4). *P < 0.05 by two-way ANOVA followed by Tukey’s test. Data represent means ± SEM.

Aldh1a3 activation is an early indicator of β-cell dedifferentiation [21]. GLB treatment resulted in the appearance of Aldh1a3-positive cells in Cyb5r3 βKO mice at approximately twice the levels of WT (Fig 3a and 3b). Aldh1a3 mRNA expression in islets was also increased by GLB treatment and further increased by Cyb5r3 deletion, mirroring the induction of Aldh1a3-positive cells (Fig 3c). FoxO1 expression was reduced by GLB treatment and loss of Cyb5r3, and further reduced by a combination of both (Fig 3d). Cyb5r3 expression was decreased by GLB administration to the same levels as Cyb5r3 βKO. Expression of β-cell markers including MafA, Pdx1, Ins2, Kir6.2 and Sur1 decreased in the GLB-treated group and in Cyb5r3 βKO compared to GLB-naïve WT mice. Levels tended to be even lower in GLB-treated Cyb5r3 βKO compared with untreated Cyb5r3 βKO. These results indicate that loss of Cyb5r3 increases β-cell dedifferentiation markers and lowers markers of differentiated β-cells.

### β-cell dedifferentiation in HFD-fed or aging Cyb5r3 βKO

**Fig 4:**
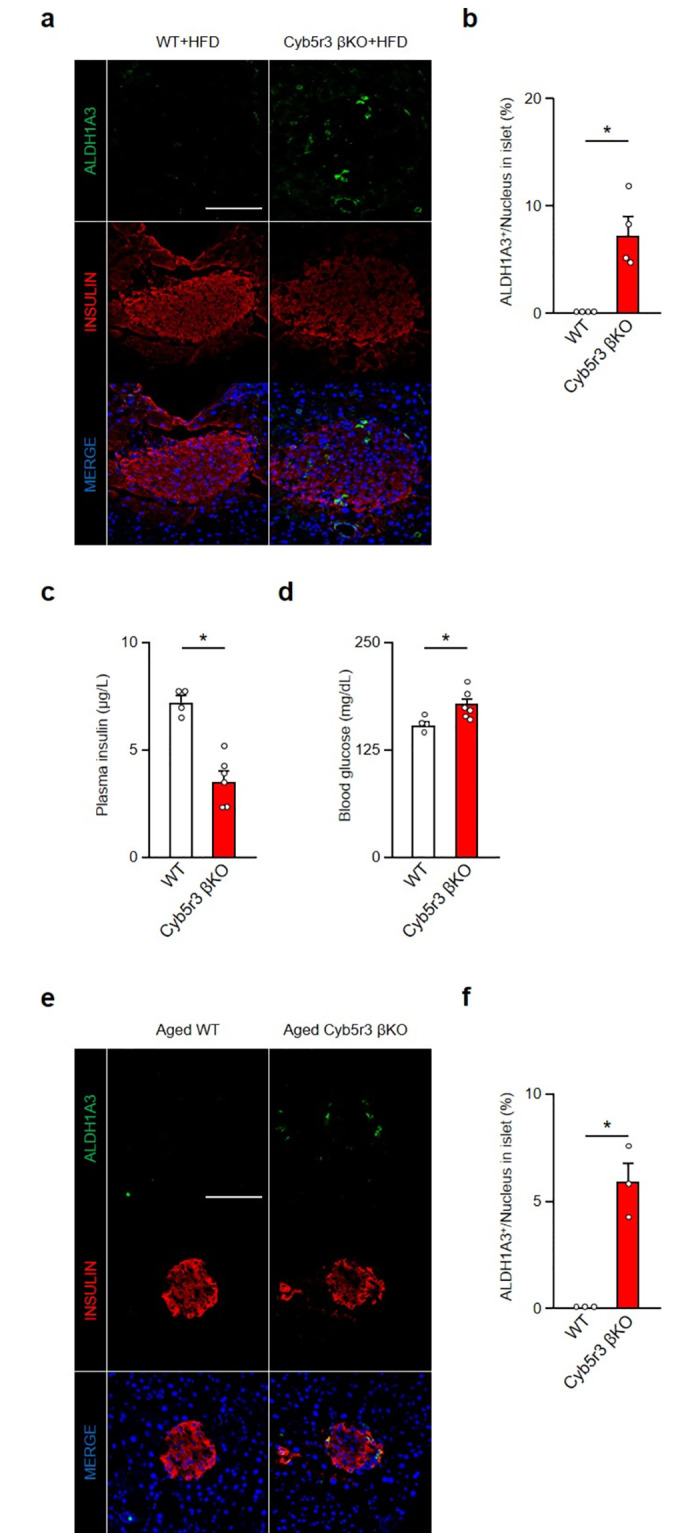
β-cell dedifferentiation in Cyb5r3 βKO also develops by HFD feeding or aging. a: Representative images from pancreatic sections of WT and Cyb5r3 βKO fed a HFD stained with Aldh1a3 (green), Insulin (red), and DAPI (blue). Scale bars: 50 μm. b: Quantification of Aldh1a3 positive cells in WT and Cyb5r3 βKO fed a HFD (n = 4). c and d: Plasma insulin (c) and blood glucose (d) levels in WT and Cyb5r3 βKO fed a HFD under free feeding condition (n = 4–6). e: Representative Aldh1a3 staining (green) in islets from pancreatic sections of aged WT and Cyb5r3 βKO (n = 3). Scale bars: 50 μm. f: Quantification of Aldh1a3 positive cells in aged WT and Cyb5r3 βKO (n = 3). *P < 0.05 by unpaired Student’s t-test. Data represent means ± SEM.

We have previously shown that loss of Cyb5r3 is associated with β-cell dedifferentiation in aging mice [11]. Here we set out to confirm those data and test the effect of a high-fat diet (HFD). Islets of HFD-fed mice did not express Aldh1a3. But 20-week HFD of Cyb5r3 βKO mice resulted in the appearance of Aldh1a3-positive islet cells (Fig 4a and 4b). Consistent with these findings, HFD-fed Cyb5r3 βKO mice showed decreased *ad lib* plasma insulin levels and increased glucose levels (Fig 4c and 4d). Aldh1a3-positive cells were absent in islets from aging WT, but present in islets from aging Cyb5r3 βKO mice (Fig 4e and 4f). These results suggest that Cyb5r3 protects against β-cell dedifferentiation caused by different agents, including GLB treatment, HFD feeding, and aging.

### Cyb5r3 activator THII does not affect β-cell dedifferentiation

**Fig 5:**
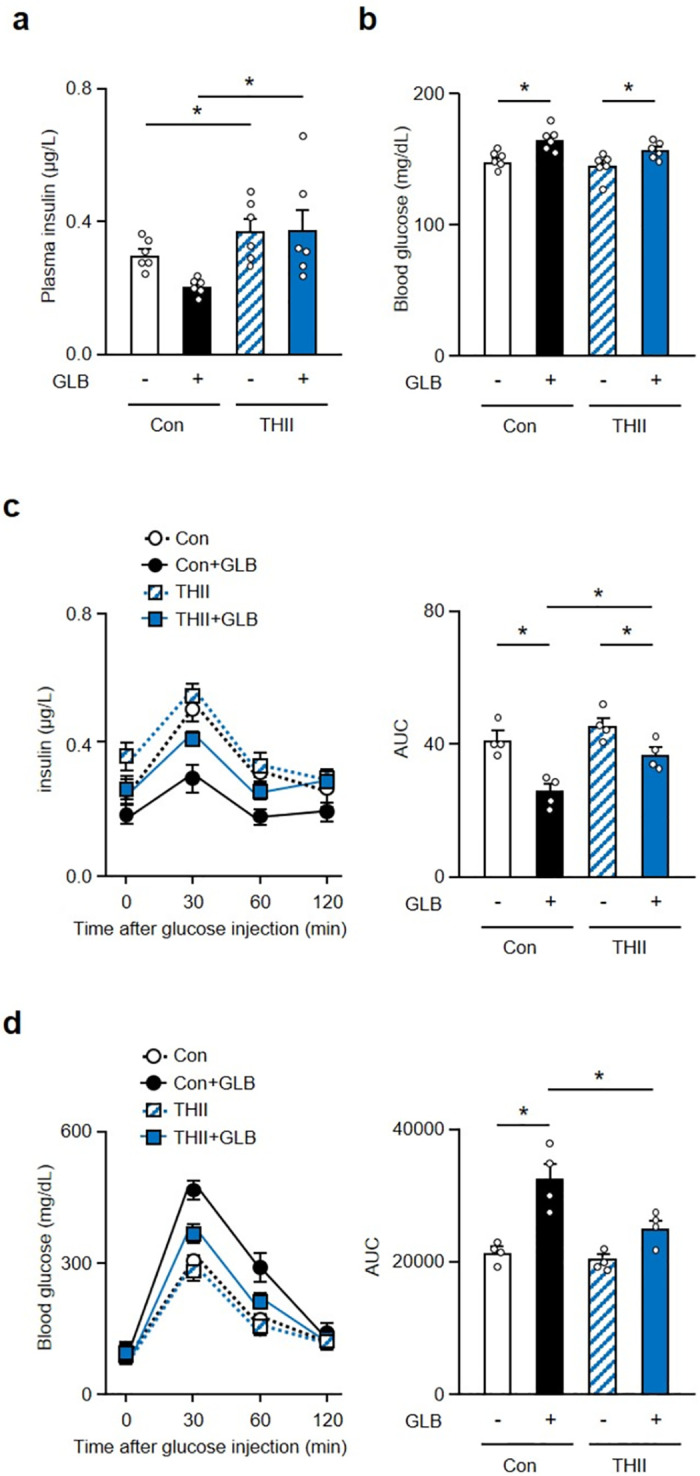
THII improves impaired glucose-induced insulin secretion in GLB treated mice. a and b: Plasma insulin (a) blood glucose (b) levels in mice treated with GLB and THII under free feeding condition (n = 4). c and d: Plasma insulin level and its area under the curve (a) and blood glucose levels and its area under the curve (d) in mice treated with GLB and THII during glucose tolerance test (n = 4). *P < 0.05 by two-way ANOVA followed by Tukey’s test. Data represent means ± SEM.

We have previously shown that the Cyb5r3 activator, THII, improves SU responsiveness in a model of SU secondary failure [9]. To determine whether THII improves β-cell function and dedifferentiation following chronic GLB treatment, we tested its effects on glucose-induced insulin secretion and Aldh1a3 expression in islets after 6-week GLB treatment. THII increased plasma insulin levels under *ad lib* conditions regardless of GLB treatment (Fig 5a). The rise of glucose levels induced by chronic GLB treatment was also reversed by THII administration (Fig 5b), as were impaired glucose-induced insulin secretion and abnormal glucose tolerance in glucose tolerance tests (Fig 5c and 5d).

**Fig 6:**
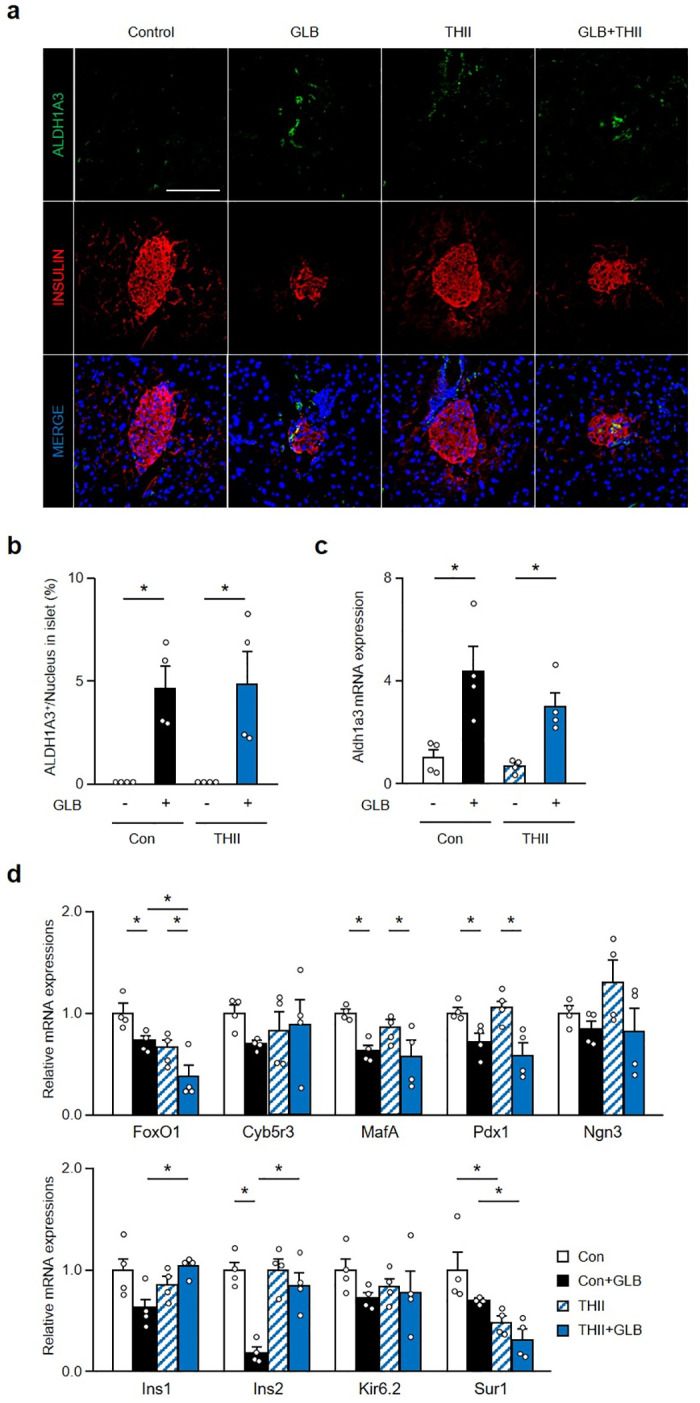
THII does not reduce β-cell dedifferentiation by chronic GLB treatment. a: Representative Aldh1a3 staining (green) in islets from pancreatic sections of mice treated with GLB and THII for 6 weeks (n = 4). Scale bars: 50 μm. b and c: Quantification of Aldh1a3 positive cells (b) and Aldh1a3 mRNA expression (c) in islets from mice treated with GLB and THII (n = 4). d: Relative mRNA expressions of FoxO1, Cyb5r3, MafA, Pdx1, Ngn3, Ins1, Ins2, Kir6.2 and Sur1 in islets from mice treated with GLB and THII (n = 4). *P < 0.05 by two-way ANOVA followed by Tukey’s test. Data represent means ± SEM.

However, while THII improved insulin secretory function following GLB treatment of mice, it did not affect the number of Aldh1a3-positive cells induced by this treatment (Fig 6a and 6b). The elevation in Aldh1a3 mRNA expression by GLB tended to be reduced by THII, but not significantly so (Fig 6c). THII did not alter expression of β-cell markers such as MafA and Pdx1, which were reduced by GLB, even as it restored Ins2 expression (Fig 6d). In addition, THII unexpectedly downregulated FoxO1 and Sur1 expression. These data indicate that Cyb5r3 activation restores impaired glucose-induced insulin secretion and insulin production associated with SU secondary failure, without reversing β-cell dedifferentiation.

## Discussion

In this study, we report that six-week GLB treatment induces β-cell dedifferentiation in a manner that is further enhanced by Cyb5r3 ablation, and that a Cyb5r3 activator restores insulin secretion but not expression of β-cell dedifferentiation markers. The data are consistent with the notion that the three steps identified in our overarching scheme of β-cell failure, decreased insulin secretion, metabolic inflexibility, and dedifferentiation [12] are mechanistically distinct and likely to respond to different treatment agents [23, 24]. β-cell dedifferentiation is the terminal stage of β-cell failure. It is preceded by decreased insulin secretion and cell fate changes. We demonstrated that these changes occur in human diabetic islets and are indeed reversible by different molecular mechanisms, consistent with the heterogeneity of the process.

The relationship between β-cell dedifferentiation and SU secondary failure is unknown. Interestingly, Amo-Shiinoki and colleagues found a correlation between disease progression and dedifferentiation in islets from type 2 diabetes patients [25], consistent with the possibility that as SU become less effective, dedifferentiation increases. In the present study, we found that exposure to GLB for six weeks induced Aldh1a3, a marker of β-cell dedifferentiation, and lowered expression of β-cell markers MafA, Pdx1, and Ins2, as it impaired insulin secretion. Thus, SU secondary failure appears to be associated with partial β-cell dedifferentiation. These effects were exacerbated by Cyb5r3 βKO, further impairing glucose-induced insulin secretion and increasing Aldh1a3 expression following chronic GLB administration to HFD-fed and in aging animals.

We have previously proposed that changes in intracellular metabolic patterns are closely related to β-cell failure [10]. In the early stages of β-cell dysfunction, FoxO1 translocates from the cytoplasm to the nucleus, presumably to protect β-cell stress by reducing lipid utilization [13, 26, 27]. Interestingly, mice homozygous for a mutant Foxo1 allele (6KR) encoding a constitutively deacetylated protein improve β-cell performance and decrease lipid oxidation [27], while β-cell specific FoxO1, FoxO3a, and FoxO4 knockout promotes lipid over carbohydrate utilization as an energy source for oxidative phosphorylation, impairs ATP production, and lowers insulin release [10]. These data are consistent with results from a metabolomic analysis of Cyb5r3 βKO islets, demonstrating decreased acetyl-CoA and TCA cycle metabolites and increased ketones [9]. We have linked these metabolomic changes with impaired Cyb5r3 regulation of glucokinase levels.

Although our data suggest that Cyb5r3 is the major factor regulating β-cell function downstream of FoxO1, FoxO1 β-cell-specific knockout mice (FoxO1 βKO) do not show major abnormalities under normal chow diet feeding condition. However, with continued metabolic stress, such as aging or repeated pregnancies, β-cells undergo dedifferentiation and hyperglycemia ensues [16]. Similarly, exogenous stresses such as HFD feeding and aging increased β-cell Aldh1a3 expression in Cyb5r3 βKO, but not in WT mice. Importantly, the β-cell dedifferentiation and altered energy metabolism observed in FoxO1 βKO closely resembles the phenotype of Cyb5r3 βKO.

The hypothesis of our study was that activation of Cyb5r3 restored both insulin secretion and β-cell dedifferentiation. But activation of Cyb5r3 ameliorated insulin secretory defects but not β-cell dedifferentiation. While THII restored Ins2 mRNA, it did not affect expression of β-cell differentiation markers, MafA and Pdx1, and reduced FoxO1 and Sur1. These results may explain why the effect of THII was limited to restoration of β-cell function. Thus, the findings are consistent with the possibility that Cyb5r3 activation can improve function in β-cells that have not yet progressed to dedifferentiation but cannot reverse dedifferentiation itself. These data are reminiscent of our analysis of master regulatory activities driving β-cell failure in human islets, showing that such master regulators have distinct effects on the three cardinal features of β-cell failure: metabolic inflexibility, cell conversion, and dedifferentiation [23].

We should point out that, although the specificity of THII for Cyb5r3 was demonstrated in our prior study, its mechanism of action is not completely understood. THII improves glucose tolerance, extends life span [28, 29], and its effects are amplified in transgenic mice expressing extra copies of Cyb5r3, consistent with its activation [29]. Its ability to reverse the impairment of insulin secretion without affecting β-cell dedifferentiation is also consistent with Cyb5r3 being its primary or even exclusive target, at least vis-à-vis the β-cell [30–32]. In future studies it will be interesting to test Cyb5r3 activators as a therapeutic target for patients suffering from β-cell dysfunction secondary to SU failure.

In conclusion, we show that chronic SU administration accelerates progression of β-cell dedifferentiation and that a Cyb5r3 activator improves insulin secretion caused by SU secondary failure without reversing β-cell dedifferentiation. These results are consistent with the view that diabetes treatment should be tailored to different phases of progression of the disease [24] and can lead to novel pharmacological approaches to preserve β-cell function and prevent diabetes progression.

## Supporting information

S1 Data (XLSX)
